# Frailty and Functional Status improvement after Skilled Nursing Facility based Post-Acute Care

**DOI:** 10.1093/geroni/igab046.2997

**Published:** 2021-12-17

**Authors:** Sandra Shi, Brianne Olivieri-Mui, Ellen McCarthy, Dae Hyun Kim

**Affiliations:** 1 Hebrew SeniorLife, Harvard Medical School, Roslindale, Massachusetts, United States; 2 Hebrew SeniorLife, roslindale, Massachusetts, United States

## Abstract

People admitted to a skilled nursing facility (SNF) for post-acute care undergo comprehensive evaluation and rehabilitation, potentially enabling prediction of future functional recovery. We identified the first SNF admission per beneficiary (n=250,159) between 07/01/2014 – 06/30/2016 in a 5% Medicare sample, using the Minimum Data Set (MDS) and the Outcome and Assessment Information Set (OASIS). Episodes were excluded for non-community discharge (n=43,397) or no OASIS admission assessment within 14 days of SNF discharge (n=77,989). A deficit accumulation Frailty Index (FI) was measured on admission MDS assessment and categorized into robust (MDS-FI<0.15), pre-frailty (MDS-FI0.15-0.24), mild frailty (MDS-FI0.25-0.34), and moderate or worse frailty (MDS-FI≥0.35). Outcomes were functional decline obtained from OASIS, readmission, or death after initiation of home care. Functional status was measured by activities of daily living from OASIS assessments. A total of 135,310 SNF episodes were matched to OASIS episodes. Of these, there were 6,472 (4.8%) robust patients, 38,923 (28.8%) pre-frail, 63,727 (47.1%) mildly frail and 26,053 (19.3%) moderately frail or worse. In a logistic regression after adjustment for OASIS admission function, compared to robust status, frailty was associated with hospital readmission or death within 30 days of OASIS admission, (mild frailty OR1.33 [95%CI 1.23-1.45] and moderate or worse OR1.81 [95%CI 1.66-1.97]). Frailty was also associated with functional decline at OASIS discharge, after adjustment for OASIS admission function (mild frailty OR1.50 [95%CI 1.38-1.63] and moderate or worse OR2.30 [95%CI 2.11-2.50]). Among those discharged from SNF with home services, a SNF-based MDS-FI is associated with increased likelihood of poor community outcomes.

